# Annotations

**Published:** 1889-04-20

**Authors:** 


### Annotations.

**Weak Hearts.**

A weak heart seems to be decidedly more practically inconvenient than a weak head. If a man or a woman be a little feeble about the region of the brain it is generally of little moment. Some post or other will be provided if the conduct be respectable; and lack of brains is too common to excite any particular attention either in the person concerned or in those about him. But a weak heart insists upon putting itself in evidence at all sorts of convenient and inconvenient times. If its possessor finds himself rather late for his morning train, and makes a "spurt" to recover lost time, the exertion is usually followed by such a "bad quarter of an hour" that he resolves in future rather to lose a dozen trains than to risk temporary suffocation or permanent syncope again. The practical evils which are associated with a feeble heart are innumerable, and will readily suggest themselves to those who possess so unsatisfactory a pumping engine. Weak hearts are by no means so common as is often supposed. Many a man who thinks he has got one is merely dyspeptic; many a woman owes her symptoms to tight-lacing or insufficient feeding. If the dyspepsia be cured, or the tight-lacing dispensed with, the symptoms of heart weakness will disappear. Even when the heart is genuinely "weak," the weakness is not always due to special disease of that organ. It may be only part of a general weakness of the whole system, which is easily curable. The late Sir Robert Christison, one of the most eminent of British physicians, used to smile at certain persons who were always complaining of weak hearts. "Gentlemen," he would say to his students when lecturing on digitalis, "gentlemen, the best tonic for a weak heart is a good brisk walk." Not a doubt of it. The majority of weak, flabby hearts are weak and flabby because every other muscle in the body is weak and flabby, and this general weakness and flabbiness is due to want of vigorous use. Exercise of the legs and back and arms gives additional and much-needed exercise to the heart, and the heart grows strong by vigorous exercise exactly as every other muscular organ does, for the heart is a muscle. If a man has no organic disease of the heart, no enlargement, and no functional disorder, plenty of brisk walking, with occasional running, will soon dispel his breathlessness and heart weakness, other things being equal. The muscular inactivity of the modern town man is the parent of more ill-health than any other single cause whatever.

**Nervous Headaches.**

There are a good many kinds of headache. In these days the nervous headache is a very distinct variety. It is generally in the front of the head, across the forehead, over the eyes. But it may be in other parts—at the top of the head, at one or both sides, at the back, or all over. It is painful, depressing, disabling. A man feels, at the height of the paroxysm, like a hunter who has galloped his legs clean off, and who could not leap a 3ft. ditch to save his life. The spur is of no use, neither is the whip. The pain in the head is worse to bear than either, and the patient will rather endure both whip and spur than make any kind of effort which will make the head pain worse. Physic by itself is of no use. There is not a single drug known to medical science which will of itself at once and permanently cure a nervous headache. On the other hand, drugs are not a ways needed. A complete change of air and circumstances will usually take away the pain in ten or twelve hours. Perfect rest, of a duration proportioned to the severity and long continuance of the symptoms, will make the cure permanent. There are, of course, methods of relieving or diminishing the pain until such time as it may be possible to obtain the complete rest. But the rest is the thing to be secured at all costs. If not, the pain goes from bad to worse, and the risk from less to greater. The final consequence it is impossible to predict, except that a breakdown is sooner or later inevitable, and the breakdown may be for a year or for a lifetime. A nervous headache is a danger-signal; if it be frequent, the danger is increased; if it be continuous, a catastrophe is imminent. The driver must put on his brake at all hazards, or he will probably soon have a leap for his life. There are very few sets of circumstances in which it is a man's duty to go on with his work when he is in this condition, at all risks. Even a threatened bankruptcy had better be risked than a threatened life. Besides, a man who is in the unyielding grip of a permanent nervous headache is not really the best judge of his own circumstances. He magnifies and distorts things amazingly. He takes counsel of his fears, and abandons his hopes and courage altogether. Rest, we repeat—immediate and sufficient rest—is the sovereign remedy. A fortnight at once may be better than a year six weeks hence.

**Progress by "Natural Selection."**

"It must not be forgotten," says Darwin, *Descent of Man*, p. 132, "that although a high standard of morality gives but a slight or no advantage to each individual man and his children over the other men of the same tribe, yet that an increase in the number of well-endowed men and an advancement in the standard of morality will certainly give an immense advantage to one tribe over another. A tribe, including many members who, from possessing in a high degree the spirit of patriotism, fidelity, obedience, courage, and sympathy, were always ready to aid one another, and to sacrifice themselves for the common good, would be victorious over most other tribes, and this would be natural selection." Darwin thoroughly appreciated the part that morality plays in the physical well-being of individuals, and in the general progress of the community. It is surprising how many, even of educated persons, fail to grasp the scientific value of pure morals. The man who makes it a part of his duty to promote with energy and enthusiasm the cause of public and private morality is looked upon as a kind of prude or precisian. We have in this country reached the high level which "permits" a man to be of good conduct without laughing at him. But though he is permitted to be honest and pure, he is not allowed to talk of the value and advantage of honesty and purity unless he happens to be a clergyman or a Sunday school teacher. Now, in plain English, that is a piece of stupid vulgarity—vulgarity, in that it is the keeping alive of a coarse and brutal prejudice which survives from coarse and brutal days. Not very long ago, moral excellence was, to a large extent, the distinguishing characteristic of religious people. Those who did not wish to be thought religious, tried to avoid the reputation of being too strictly moral. An "easy latitude" was the fashionable state of mind in "the days of Queen Anne" and later. But science is nothing if not positive. It demands facts for observation, and facts for recording. Science has discovered that a rigid morality amounting almost to "Puritanism," is the very prime condition and necessity of perfect health, both in the individual and in the community. This is not the least surprising to persons imbued in any degree with the philosophic spirit. Religion, philosophy, and science all spring from a common root and a common soil—the intellectual and moral faculties of man. It would be strange, indeed, if, springing from the same soil and the same root, they should produce fruits mutually contradictory and mutually destructive. In ultimate analysis we see that Cardinal Manning and Mr. Darwin are entirely at one as to the value of good conduct. As Darwin says elsewhere, "To do good unto others—to do unto others as ye would that they should do unto you—is the foundation-stone of morality."

